# Quantification of Phenolic Compounds in Olive Oils by Near-Infrared Spectroscopy and Multiple Regression: Effects of Cultivar, Hydroxytyrosol Supplementation, and Deep-Frying

**DOI:** 10.3390/antiox14060672

**Published:** 2025-05-31

**Authors:** Taha Mehany, José M. González-Sáiz, Consuelo Pizarro

**Affiliations:** Department of Chemistry, University of La Rioja, 26006 Logroño, Spain; taha.abdellatif@unirioja.es (T.M.); josemaria.gonzalez@unirioja.es (J.M.G.-S.)

**Keywords:** antioxidants, caffeic acid, calibration models, EVOO, hydroxytyrosol, ligstroside aglycone, lipid oxidation, oleuropein, oleocanthal, oleacein, phenols, SELECT-OLS

## Abstract

Near-infrared (NIR) spectroscopy, combined with multivariate calibration techniques such as stepwise decorrelation of variables (SELECT) and ordinary least squares (OLS) regression, was used to develop robust, reduced-spectrum regression models for quantifying key phenolic compound markers in various olive oils. These oils included nine extra virgin olive oil (EVOO) varieties, refined olive oil (ROO) blended with virgin olive oil (VOO) or EVOO, and pomace olive oil, both with and without hydroxytyrosol (HTyr) supplementation. Olive oils were analyzed before and after deep frying. The results show that HTyr ranged from 7.28 mg/kg in Manzanilla (lowest) to 21.43 mg/kg in Royuela (highest). Tyrosol (Tyr) varied from 5.87 mg/kg in Royuela (lowest) to 14.86 mg/kg in Hojiblanca (highest). Similar trends were observed in all phenolic fractions across olive oil cultivars before and after deep-frying. HTyr supplementation significantly increased both HTyr and Tyr levels in non-fried and fried supplemented oils, with HTyr rising from single digits in some controls (around 0 mg/kg) to over 300 mg/kg in most of the supplemented samples. SELECT efficiently reduced redundancy by selecting the most vital wavelengths and thus significantly improved the regression models for key phenolic compounds, including HTyr, Tyr, caffeic acid, decarboxymethyl ligstroside aglycone in dialdehyde form (oleocanthal), decarboxymethyl oleuropein aglycone in dialdehyde form (oleacein), homovanillic acid, pinoresinol, oleuropein aglycone in oxidized aldehyde and hydroxylic form (OAOAH), ligstroside aglycone in oxidized aldehyde and hydroxylic form (LAOAH), and total phenolic content (TPC), achieving correlation coefficients (R) of 0.91–0.98. The SELECT-OLS method generated highly predictive models with minimal complexity, using at most 30 wavelengths out of 700. The number of decorrelated predictors varied, at 12, 14, 15, 30, 30, 21, 30, 30, 30, and 18 for HTyr, Tyr, caffeic acid, oleocanthal, oleacein, homovanillic acid, pinoresinol, OAOAH, LAOAH, and TPC, respectively, demonstrating the adaptability of the SELECT-OLS approach to different spectral patterns. These reliable calibration models enabled online and routine quantification of phenolic compounds in EVOO, VOO, ROO, including both non-fried and fried as well as supplemented and non-supplemented samples. They performed well across eight deep-frying conditions (3–6 h at 170–210 °C). Implementing an NIR instrument with optimized variable selection would simplify spectral analysis and reduce costs. The developed models all demonstrated strong predictive performance, with low leave-one-out mean prediction errors (LOOMPEs) with values of 15.69, 8.47, 3.64, 9.18, 16.71, 3.26, 8.57, 13.56, 56.36, and 82.38 mg/kg for HTyr, Tyr, caffeic acid, oleocanthal, oleacein, homovanillic acid, pinoresinol, OAOAH, LAOAH, and TPC, respectively. These results confirm that NIR spectroscopy combined with SELECT-OLS is a feasible, rapid, non-destructive, and eco-friendly tool for the reliable evaluation and quantification of phenolic content in edible oils.

## 1. Introduction

Virgin olive oil (VOO) is a key component of the Mediterranean diet (MD), renowned for its nutritional value and health benefits. The MD, rich in protective nutrients and bioactive compounds, has been linked to the prevention of various diseases, including obesity and cancer [1]. In addition, VOO is more resistant to oxidation than most edible oils, offering a relatively long shelf life (12–18 months). This stability is due to its high antioxidant content, particularly phenolic compounds, and its low levels of polyunsaturated fatty acids [2]. Among the different categories of olive oil, extra virgin olive oil (EVOO) stands out for its superior composition and sensory attributes, evaluated by recognized panels [3]. Regular EVOO consumption is associated with a lower risk of chronic diseases such as cardiovascular disease, stroke, type 2 diabetes, metabolic syndrome, cognitive decline, and certain cancers, including breast and colorectal cancer. Additionally, it has been shown to reduce obesity risk, prevent weight gain, and improve overall longevity, underscoring its importance in dietary recommendations [4].

Olive oil is primarily composed of triacylglycerols, with a minor fraction (0.5–1.0%) consisting of non-glyceridic compounds, including over 30 phenolic compounds that enhance oxidative stability. A strong correlation exists between phenolic content and EVOO’s resistance to rancidity, with extra virgin olive oils consistently exhibiting higher phenol levels than refined oils [5,6,7]. EVOO’s health benefits are largely attributed to its phenolic content and its fatty acid composition [8]. The type and concentration of phenols depend on factors such as olive variety, cultivation, harvesting, and processing methods [9]. In addition to their biological activity, these compounds contribute to EVOO’s distinctive sensory characteristics [10,11]. This minor fraction also contains free fatty acids, tocopherols, sterols, phospholipids, waxes, squalene, hydrocarbons, and volatile compounds, some of which influence both flavor and health properties. Phenolic compounds, in particular, are responsible for EVOO’s characteristic bitterness and pungency while enhancing its antioxidant capacity. They also inhibit low-density lipoprotein (LDL) oxidation, a key factor in atherosclerosis, and provide various other protective health effects [12,13]. Besides phenolic compounds, the unsaturated fatty acids (UFAs) in VOO, particularly oleic and linoleic acids, have been shown to impact cancer development and metastasis by suppressing the overexpression of the oncogene HER2 [14].

Phenolic compound extraction from olive oil using traditional methods such as liquid–liquid extraction (LLE) and solid-phase extraction (SPE) has several disadvantages. LLE often requires large volumes of toxic organic solvents, is time-consuming, and can lead to emulsion formation, complicating phase separation. SPE, while reducing solvent use, is associated with high operational costs, limited sample capacity, and potential analyte loss during extraction. In contrast, newer extraction techniques—such as ultrasound-assisted extraction (UAE), microwave-assisted extraction (MAE), and miniaturized solvent techniques—offer several advantages, including reduced solvent consumption, shorter extraction times, improved extraction efficiency, and lower environmental impact. These modern approaches can enhance reproducibility and are more suitable for routine analysis and high-throughput applications. Moreover, numerous methods for determining phenolic compounds in olive oil have been reported, including high-performance liquid chromatography (HPLC), LC-MS, HPLC coupled with fluorescence detection (FLD) or photodiode array (PDA), nuclear magnetic resonance (NMR), mass spectrometry (MS), and, more recently, time-of-flight mass spectrometry (TOF-MS), gas chromatography–mass spectrometry (GC-MS), colorimetric assay (Folin–Ciocalteu method), and capillary electrophoresis (CE) [15,16,17,18,19,20]. While these techniques are effective and offer highly sensitive and specific tools for identifying and quantifying phenolic compounds in complex matrices like olive oil, they have several drawbacks. They are often time-consuming due to extensive sample preparation and long analysis times, making them inefficient for rapid screening. Additionally, they are costly, requiring specialized equipment, solvents, and reagents. Complex sample preparation, solvent usage, and environmental concerns further add to their limitations. Some methods struggle with detecting low-concentration phenolic compounds, while high-temperature techniques like GC can lead to compound degradation. Moreover, these techniques require skilled operators for precise instrument handling and data interpretation.

To address these limitations, non-destructive vibrational spectroscopic techniques such as near infrared (NIR), mid infrared (MIR), and Raman spectroscopy have emerged as efficient alternatives for analyzing olive oils. These methods enable the rapid and accurate identification of key bioactive compounds, including unsaturated fatty acids, phenolic compounds, and antioxidants, surpassing traditional approaches [20,21,22,23]. Advances in spectroscopy, data processing, and chemometric techniques have further enhanced detection accuracy. Vibrational spectroscopy provides precise measurement of compound concentrations and structural variations, offering a robust scientific foundation for quality certification and functional assessment of olive oil [20,24]. Among spectroscopic techniques, near-infrared (NIR) spectroscopy stands out for its speed, environmental friendliness, and non-destructive nature, making it highly suitable for both qualitative and quantitative analysis of olive oil. However, it does have some limitations, including lower sensitivity and specificity compared with traditional chromatographic methods. The accuracy of NIR spectroscopy relies heavily on the development of robust calibration models, as overlapping spectral bands can complicate the precise quantification of chemically similar compounds within complex matrices. Therefore, the development of a reliable and robust NIR calibration model is of significant importance for improving the analytical performance and applicability of this technique in olive oil evaluation [25,26,27,28]. NIR is a spectroscopic technique that operates on the principle of molecular vibrations. When NIR light, typically within the 780–2500 nm wavelength range, interacts with a sample, specific molecules absorb light energy at characteristic wavelengths. This absorption induces transitions in vibrational energy levels, corresponding to the vibrational modes of chemical bonds such as C–H, N–H, and O–H. By analyzing the intensity of these absorption peaks, valuable insights into the molecular composition of the sample can be obtained [29].

Artificial intelligence, chemometrics, machine learning, and deep learning are promising tools that lead to a clearer and better understanding of data, due to their ability to model complex datasets and classify unknown samples [30]. However, the accuracy of these models can be affected by redundant variables, irrelevant information, and instrumental noise [31,32]. NIR spectroscopy, widely used across various industries, benefits from variable selection to enhance precision and interpretability. Identifying relevant wavelengths is crucial for improving predictive models [33]. Variable selection plays a crucial role in multivariate regression and has become an essential tool across various research fields [34]. 

This study employed SELECT as a chemometric tool for variable selection on NIR spectra of various olive oils to extract a minimal yet highly informative set of predictors [35,36,37]. By iteratively reducing collinearity through the SELECT algorithms, SELECT enhances model performance and interpretability. This approach facilitates the use of linear regression models, which, despite their simplicity and ease of interpretation, are particularly sensitive to collinearity among regressors. Furthermore, ordinary least squares (OLS) regression is a commonly used statistical technique for estimating relationships between variables by minimizing errors in a least-squares sense. It plays a key role in multivariate calibration and predictive modeling, operating under assumptions of linearity, independence, and normality of residuals [38,39].

This study aims to develop a novel, unified model for quantifying phenolic compounds in various olive oil types. This study evaluated phenolic compounds in nine EVOO cultivars and three olive oil blends (virgin or EVOO mixed with refined oils) under various treatments, including HTyr supplementation and deep-frying. Both fried and non-fried samples were analyzed. Phenolic quantification was performed using HPLC, and predictive modeling was developed using NIR spectroscopy combined with SELECT and OLS regression methods.

## 2. Materials and Methods

### 2.1. Materials

Olive fruit extract (OFE) enriched mainly with hydroxytyrosol (HTyr) and its derivatives was obtained from Natac BioTech, Madrid, Spain. The chemicals and reagents used in this study included phosphoric acid (H_3_PO_4_) (49–51%), purchased from Sigma-Aldrich (Saint Louis, MO, USA). HPLC-grade syringic acid (≥97% purity) and tyrosol (≥98% purity) were obtained from Sigma-Aldrich Chemie GmbH (Steinheim, Germany). LC-MS-grade methanol (≥99.9% purity) and acetonitrile (100% purity) were supplied by Fisher Scientific Ltd. (Loughborough, UK). Ultrapure water was sourced from a Milli-Q system (Millipore, Bedford, MA, USA).

### 2.2. Olive Oil Sampling and Experimental Design

The present study examined four categories of olive oils. The olive oils included nine EVOOs from different Spanish varieties, i.e., Picual, Cornicabra, Empeltre, Arbequina, Hojiblanca, Manzanilla Cacereña, Royuela/Arróniz, Koroneiki, and Arbosana, sourced from various Spanish producers. Additionally, an EVOO blended with refined olive oil (ROO) and labeled olive oil 1° (maximum acidity 1% as oleic acid), a refined olive oil mixed with EVOO, known as pomace olive oil or as Orujo oil in Spain, and a virgin olive oil blended with ROO, labeled olive oil 0.4° (maximum acidity 0.4% as oleic acid). Table 1 shows the experimental design (2^3^) methodology including various olive oil types supplemented with olive fruit extract under different deep-frying conditions. Eleven samples were analyzed for each olive oil category, including (1) Control 1 (original, non-fried olive oil), (2) Control 2 (a mixture of non-fried original olive oil and olive oil supplemented with HTyr), (3) supplemented non-fried olive oil, and (4) eight deep-fried (D-F) samples under full factorial experimental design (Table 1) subjected to different conditions such as time, temperature, and polyphenol addition. In total, 132 samples were evaluated, covering both control oils and those processed under deep-frying experimental conditions. In addition, Table 2 represents the sampling design for the various olive oil categories used in this study.

### 2.3. Supplementation Procedure of Various Olive Oils with HTyr Extract

Olive oils were supplemented with HTyr-rich OFE to evaluate the effect of supplementation during deep-frying. The process involved enriching base oils (EVOO or other olive oil types) to create a polyphenol-rich supplemented oil, which was then blended with the original oil to form Control 2.

**Figure 1:**
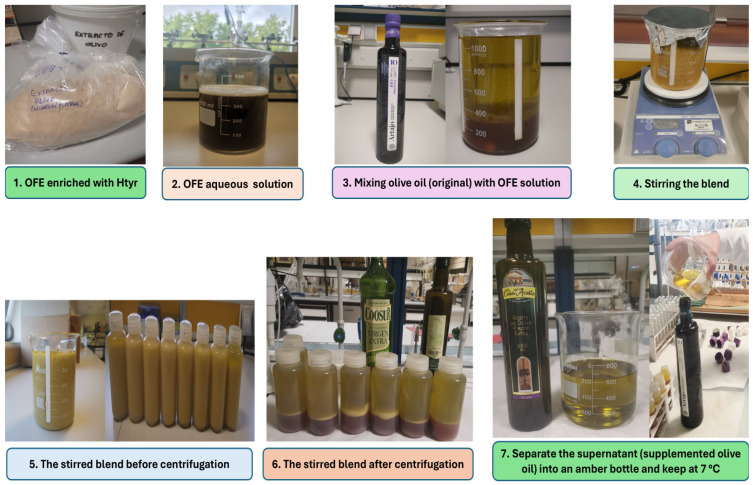
The supplementation process of olive oil with olive fruit extract enriches it with hydroxytyrosol (HTyr) and its derivatives.

In detail, 40 g of OFE extract was added to 400 g of H_2_O (10% *w*/*v*). Then, the solution was stirred using a magnetic stirrer (IKA-WERKE, Staufen, Germany) at room temperature (RT) for 30 min. Subsequently, 200 g of this aqueous extract solution was mixed with 500 g of olive oil (2 OFE aqueous solution: 5 olive oil *w*/*w*), and the mixture was stirred mechanically at RT for 60 min. The prepared solution was then centrifuged (9961× *g*/20 min using a Sorvall RC-6 Plus device, Thermo Scientific, Dreieich, Germany) [40]. The supplemented oil (supernatant phase) was stored at 7 °C ± 2 in an amber container for further analysis. In this regard, Figure 1 illustrates the supplementation process of olive oil with olive fruit extract, which enriches it with HTyr and its derivatives.

### 2.4. Deep-Frying Experiments

Various categories of olive oils (supplemented or not with HTyr extract) were heated using a Soxhlet heating apparatus (SELECTA, Barcelona, Spain). In each deep-frying experiment, 400 mL of oil was placed in the fryer and continuously heated at 170 ± 10 °C for 3 or 6 h or at 210 ± 10 °C for 3 or 6 h. After frying under different conditions including varying the oil type, duration, temperature, and exogenous polyphenol addition, 400 mL of oil was collected in standard amber glass containers for further analysis of phenolic compounds using HPLC as a reference method, and spectra were recorded using NIR spectroscopy (132 samples in triplicates). The samples were stored at 5 °C in the dark to prevent further oxidation before analysis.

### 2.5. Extraction of Phenolic Compounds from Olive Oil Samples and Their Quantification by HPLC Analysis

For extraction and analyses of phenolic compounds from olive oil samples, the protocol of the International Olive Council [41] was followed. About 2.0 g of each deep-fried olive oil sample and non-fried samples (controls) were placed in 10 mL screw-cap test tubes. Then, 1 mL of the internal standard solution (syringic acid) was added to the previously weighed sample. The tubes were sealed with screw caps and shaken vigorously for exactly 30 s at RT using a shaker (Heidolph, D-91126, Schwabach, Germany). Next, 5 mL of the methanol/water (80/20, *v*/*v*) extraction solution was added to each tube. Then, the samples were shaken robustly again for 1 min. Additionally, the samples were sonicated in an ultrasonic bath for 15 min at 30 °C (Ultrasons 00-A, 50/60 Hz, 360 W, J. P. SELECTA, Barcelona, Spain) with the use of a shaker (Heidolph, RZR1, Schwabach, Germany) and a water bath to adjust the extraction temperature to 30 °C (Julabo, F25, Seelbach, Germany). Finally, the ultrasonicated and extracted samples were centrifuged (Eppendorf 5403 Refrigerated Centrifuge, Hamburg, Germany) at 4193× *g*/25 min. Then, an aliquot from the supernatant phase was filtered through a 5 mL plastic syringe, with a 0.45 µm PVDF filter (Millex HV, Merck Millipore Ltd., Cork, Ireland), for further injection for HPLC analysis.

HPLC analysis was performed using a Hewlett Packard 1100 series system (Agilent Technologies, Waldbronn, Germany), equipped with a high-pressure gradient pump, a photodiode array detector (DAD), an autosampler, and a degasser. Separation was carried out on a Spherisorb octadecyl silyl (ODS) column (250 mm × 4.6 mm ID, 5.0 μm particle size, Waters, Dublin, Ireland). The mobile phase consisted of a mixture of 0.2% H_3_PO_4_ in water (*v*/*v*), methanol, and acetonitrile (96/2/2, *v*/*v*/*v*), using gradient elution. To determine response factors (RF), 20 µL of the external calibration standard solution containing tyrosol and syringic acid was injected, allowing the calculation of the relative response factor (RRF) for syringic acid and tyrosol. Following calibration, a 20 µL aliquot of each sample was injected into the HPLC system and analyzed in triplicate. The chromatograms were recorded at 280 nm. The polyphenol content, expressed in mg/kg, was calculated using the following equation:

(1) $$\text{Polyphenols content}\left(\frac{\mathrm{mg}}{\mathrm{kg}}\right)=\frac{\left(\Sigma \mathrm{Area}\right)\times 1.000\times \frac{\mathrm{RRFsyr}}{\mathrm{tyr}}\times \left(\text{Weight of syringic acid}\right)}{\left(\text{Area syringic acid}\right)\times \left(\text{Weight of sample}\right)}$$

where RRF is the multiplication coefficient of (Syr) syringic acid/(Tyr) tyrosol.

**Figure 2:**
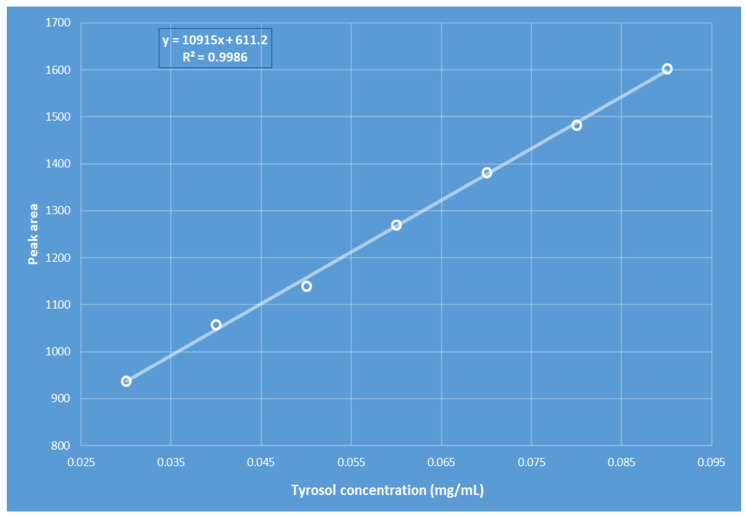
Tyrosol calibration curve used for validation of the HPLC method.

A standard stock solution of tyrosol (1 mg/mL) was prepared by dissolving 10 mg of tyrosol in 10 mL of an 80:20 (*v*/*v*) methanol/water mixture. This solution was then used to generate a series of standard concentrations ranging from 0.030 to 0.090 mg/mL. To construct the HPLC calibration curve, the ratio of the tyrosol peak area to its corresponding concentration was plotted (Figure 2). A linear relationship was observed between the peak area and the tyrosol concentration within this range, described by the regression equation y = 10915x + 611.2 with an R^2^ value of 0.9986, where *x* denotes tyrosol concentration (mg/mL) and *y* represents the peak area. The calculated limits of detection (LOD) and quantification (LOQ) were 0.0098 mg/kg and 0.0298 mg/kg, respectively. For assessment of accuracy (trueness and precision), each oil sample was analyzed in triplicate. Standard deviation values were used to evaluate the consistency of measurements across repeated samplings of the same homogeneous sample, confirming the method’s precision, reproducibility, repeatability, and overall reliability.

### 2.6. Near Infrared (NIR) Spectroscopy

A total of 396 spectra were recorded from 132 olive oil samples, each analyzed in triplicate using NIR spectroscopy. Before spectral acquisition, samples were centrifuged (Sorvall RC-6 Plus, Dreieich, Germany) at 20,000 rpm for 30 min to remove particles and dispersed water droplets, minimizing light-scattering effects. NIR spectra were collected using a Foss NIRSystems 5000 spectrophotometer (Foss NIRSystems, Silver Spring, MD, USA), equipped with a thermostated liquid analyzer module and a Suprasil quartz flow cell. The spectral acquisition parameters included an optical path length of 10 mm; 700 wavelengths ranging from 1100 to 2498 nm were recorded at spectral resolution of 2 nm and 32 scans per spectrum.

### 2.7. Feature Variables Selection by SELECT and OLS Regression Model Development for Phenolic Compounds Quantification

To enhance data quality, preprocessing techniques such as column autoscaling were applied. The content of phenolic compounds in extra virgin and refined oil blends with virgin or extra virgin olive oils was quantified using OLS regression after employing SELECT algorithms for feature variables selection. Selection of variables and development of the linear regression model were conducted using V-PARVUS software (PARVUS2011, Michele Forina, Genoa, Italy), with the SELECT algorithm identifying a subset of decorrelated, significant variables to optimize the regression models.

The SELECT algorithm iteratively selects the most influential wavelength while minimizing redundancy and collinearity, improving predictive accuracy. SELECT was employed to identify the most relevant predictors for NIR spectroscopy. SELECT generates a set of decorrelated variables. It identifies the variable with the highest weight, selects it, and then removes its correlation with the remaining variables. This process continues, selecting variables with the highest weights until a predefined number of variables is reached or the weight falls below a specified threshold [37]. Eliminating unnecessary predictors is crucial, as redundant variables can weaken the predictive performance of the regression model [36].

OLS regression models were evaluated using leave-one-out (LOO) cross-validation metrics, including residual variance, residual standard deviation, explained variance, and mean prediction error. The most robust model was identified as the one with the lowest LOO mean prediction error. LOO residual variance and standard deviation were used as error metrics, while LOO explained variance reflected the proportion of variance captured. LOO mean prediction error represented the model’s average prediction error during cross-validation. To prevent overfitting, the optimal number of decorrelated predictors in the SELECT-OLS model was determined through complete validation, ensuring unbiased predictive accuracy. By ranking variables based on weights and selection frequency, the SELECT algorithm further refined the regression models, improving both interpretability and predictive performance.

## 3. Results and Discussion

### 3.1. Changes in Phenolic Compounds Content by HPLC Across Various Olive Oil Cultivars, Hydroxytyrosol Supplementation, and Deep-Frying

As shown in the results presented in Table 3, the changes in phenolic compounds across nine different EVOO cultivars—Picual, Cornicabra, Empeltre, Arbequina, Hojiblanca, Manzanilla Cacereña, Royuela/Arróniz, Koroneiki, and Arbosana—were studied. Additionally, one EVOO mixed with refined olive oil (1° acidity), one pomace olive oil mixed with EVOO, and one virgin olive oil mixed with refined olive oil (0.4° acidity) were included, along with hydroxytyrosol supplementation and deep-frying treatments.

Regarding the cultivars, there were clear variations in hydroxytyrosol content across the different cultivars. Royuela showed the highest level (21.43 mg/kg), while Manzanilla showed the lowest (7.28 mg/kg). For tyrosol, Hojiblanca had the highest value (14.86 mg/kg), whereas Royuela showed the lowest (5.87 mg/kg).

As presented in Table 2, the results of the full factorial experimental design demonstrate clear quantitative differences in phenolic compound concentrations among the differently treated samples. Deep-frying significantly affected the levels of specific phenols, with a general trend of degradation observed. Moreover, distinct EVOO cultivars showed notable variations in their phenolic profiles. For example, original samples from Royuela, Arbosana, and Empeltre cultivars showed high total phenolic contents of 400.63, 393.00, and 337.00 mg/kg, respectively, whereas refined samples such as Orujo and 0.4° olive oil contained significantly lower levels, at 3.89 and 26.50 mg/kg, respectively, confirming both the impact of thermal processing, refining, and cultivar differences.

These trends were also observed in all phenolic fractions in EVOO cultivars before deep frying, probably due to differences in environmental conditions, agricultural practices, and the specific cultivar characteristics, which significantly affect phenolic content in terms of both quantity and antioxidant quality.

From the same table, it can be observed that exogenous hydroxytyrosol supplementation dramatically increased the phenolic content in the supplemented oils and Control 2 samples, as well as in the deep-fried samples supplemented with hydroxytyrosol extract, especially increasing the content of hydroxytyrosol and tyrosol. Moreover, HTyr and Tyr decreased gradually with the progress of deep-frying. This extract also played a significant role in improving the stability and antioxidant potential of olive oil before and after deep frying.

### 3.2. NIR Spectra Interpretation of Oil Samples

**Figure 3:**
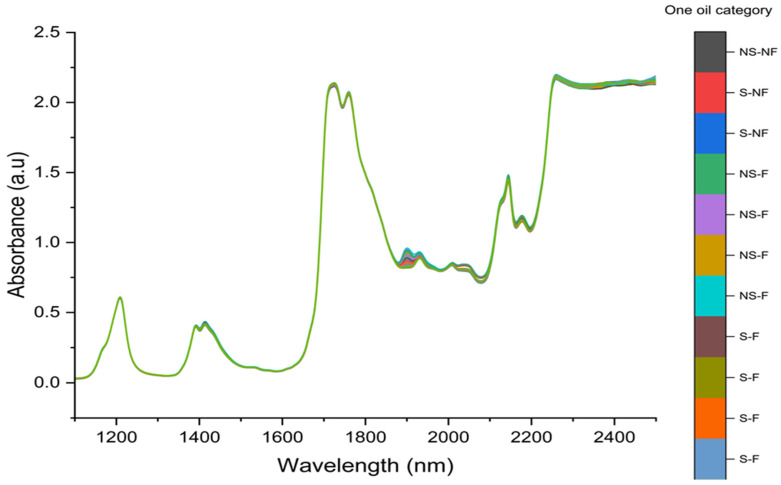
NIR spectra of different categories of supplemented olive oils with HTyr under frying conditions, compared with non-fried and non-supplemented olive oils with HTyr. NS-NF: Non-supplemented, non-fried; S-NF: Supplemented, non-fried; NS-F: Non-supplemented, fried; S-F: Supplemented, fried.

In this study, 396 spectra from 132 different olive oil types were recorded using NIR spectroscopy to quantify phenolic compounds, covering hydroxytyrosol supplementation and deep-frying as well as non-fried and non-supplemented oils. The findings included prominent peaks at 1204, 1208, 1210, 1212, 1214, and 1216 nm, which are useful for evaluating quality parameters, including free fatty acid content. Additionally, significant absorption at 1388, 1390, 1392, 1394, and 1396 nm was observed; these spectral regions are linked to O-H combination bands and are valuable for quality assessment (Figure 3).

The spectral range of 1350–1570 nm proved particularly effective for distinguishing different olive oils, aiding in authentication and quality control by identifying original olive oil and detecting primary oxidation compounds. Higher absorbance was recorded at 1408, 1410, 1412, 1414, 1416, and 1418 nm, with particularly strong signals at 1414 and 1416 nm, corresponding to overtones of C-H and O-H bonds (Figure 3).

Furthermore, the NIR spectra of various olive oils exhibited significant absorption around 1724 nm due to the first overtone of C-H vibrations. Similarly, absorption at 1760 nm was linked to the first overtone of C-H vibrations and lipid oxidation, enabling the detection of primary oxidation products. Higher absorbance was also detected at 1860 and 1862 nm, particularly at 1860 nm. Additionally, strong absorbance was noted at 1890, 1892, 1894, 1896, 1898, 1900, 1902, and 1904 nm, with a prominent peak at 1900 nm, indicating oxidation and degradation.

High absorbance around 2144 nm corresponded to C=O stretching (carbonyl compounds), indicating the formation of aldehydes and ketones from lipid degradation. Similarly, a distinct absorption peak at approximately 2226 nm was linked to O-H and C-H combination bands, making it valuable for assessing hydrolysis and secondary oxidation. Significant absorption at 2250, 2252, 2254, and particularly at 2256 nm varied among the samples and was useful for distinguishing oxidation stability while also associated with O-H and C-H combination bands.

The 1700–2500 nm region was strongly correlated with lipid oxidation and hydrolytic degradation (Figure 1). NIR devices also demonstrated effectiveness in classifying EVOO based on spectral variations. These findings confirm the broad applicability of NIR spectroscopy for ensuring the authenticity, freshness, and overall quality of EVOO [42,43,44].

These findings highlight the importance of selecting stable olive oils for frying and employing NIR spectroscopy for real-time monitoring to ensure the safety and quality of fried extra virgin olive oils, virgin olive oils, and their blends. NIR not only serves as a valuable tool for tracking EVOO oxidation during deep frying, as specific wavelength regions correspond to compounds formed during oil degradation, but it can also be used for rapid quantification of phenolic compounds in olive oils instead of conventional methods like Folin–Ciocalteu and HPLC approaches. This enables effective assessment of oil quality and safety. As shown in Table 4, these critical wavelengths play a key role in monitoring oil degradation and quality indices. Oxidation results in the formation of peroxides, aldehydes, and other degradation products that significantly impact the oil’s quality, taste, and safety during deep frying.

### 3.3. SELECT-OLS Models for Quantification of Phenolic Compounds

The findings reveal that the SELECT approach identified only 12 key infrared (IR) wavelengths out of 700 for quantifying hydroxytyrosol (HTyr) across various olive oil categories (Table 5). Among these, 1962 nm ranked first, followed by 1856 nm, which ranked second. The 1962 nm wavelength exhibited strong absorbance in most EVOO varieties supplemented with HTyr and in non-fried olive oil. Comparing its absorption intensity with pure EVOO spectra can help detect dilution with olive fruit extract-supplemented oils. This suggests that NIR and SELECT can effectively distinguish between supplemented/non-fried olive oil and deep-fried samples.

EVOO samples subjected to continuous deep frying at 210 °C for 6 h showed significantly lower HTyr levels than those fried at 170 °C for 3 h. Higher HTyr content was observed in HTyr-supplemented samples, emphasizing the protective effect of lower frying temperatures against oxidation [51]. Moreover, HTyr in supplemented oils maintained a more stable phenolic composition during prolonged high-temperature frying than other phenolic fractions.

The SELECT algorithm identified distinct wavelengths, highlighting that spectral variations and oxidation features depended on EVOO variety, supplementation, frying conditions (temperature/duration), and HTyr stability. The optimization process achieved remarkable data compression—selecting only 12 wavelengths from 700 predictors—to model HTyr content in EVOO, refined VOO, and mixed EVOO, whether supplemented or not or deep-fried or not.

**Figure 4:**
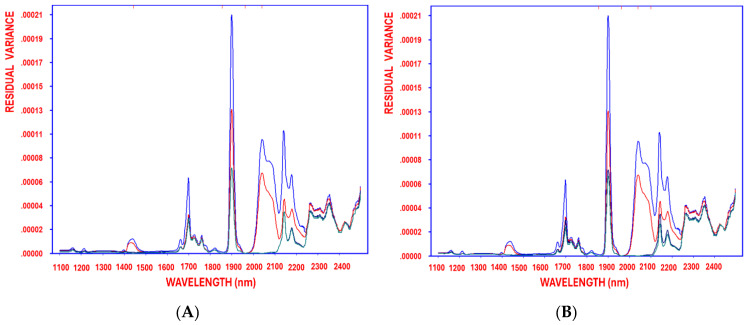

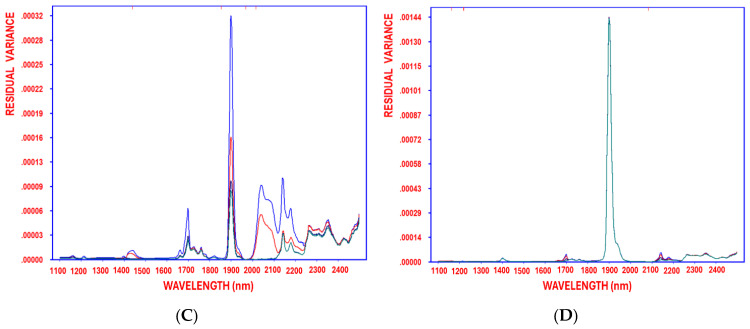
Residual variance of the variables decorrelated by SELECT after 1, 2, 3, and 4 (blue, red, turquoise, green, respectively) selections obtained when working on auto-scaled NIR spectra, using hydroxytyrosol (**A**), tyrosol (**B**), caffeic acid (**C**), and oleocanthal (**D**) as response variables.

The high reliability and robustness of the resulting OLS models underscore the effectiveness of SELECT-OLS for feature selection and correction. The progressive reduction in residual variance, tracked from the initial stage through successive decorrelation cycles, led to an optimal model with negligible residual variance (Figure 4A–D), using hydroxytyrosol, tyrosol, caffeic acid, and oleocanthal as response variables, respectively. The SELECT algorithms output the retained original variables, demonstrating their efficiency in refining predictive models [36,37].

Regarding tyrosol, the findings indicate that the SELECT approach identified 14 key spectral variables out of 700 for predicting tyrosol content across various olive oil categories, considering hydroxytyrosol supplementation and different deep-frying conditions (Table 6). Among these, 1962 nm was most significant, followed by 1856 nm. The results show that the 1962 nm wavelength exhibited high absorbance in most EVOO varieties supplemented with HTyr and in blends of original olive oil with HTyr-supplemented olive oil. This suggests that NIR and SELECT can effectively distinguish between supplemented and non-supplemented olive oils, as well as between non-fried and deep-fried samples.

Furthermore, EVOO samples subjected to continuous deep frying at 210 °C for 6 h exhibited significantly lower tyrosol levels compared with those fried at 170 °C for 3 h, indicating the impact of frying temperature and duration on tyrosol degradation. A similar trend was observed in HTyr-supplemented samples, highlighting the protective effect of HTyr extract in preserving tyrosol content in olive oil. Additionally, pomace olive oil and olive oil with 0.4° acidity contained zero tyrosol, indicating their low phenolic compound content compared with EVOO varieties.

In addition, the SELECT approach identified 15 significant wavelengths out of 700 for quantifying caffeic acid (Table 7), with the 1968 nm wavelength selected as the first-order variable. The 1968 nm wavelength is associated with overtones and combination bands of molecular vibrations, primarily related to C-H, O-H, and aromatic functional groups found in phenolic compounds, including caffeic acid in olive oil. Caffeic acid, a key phenolic compound, contributes to the antioxidant properties, bitterness, and stability of olive oil [52,53]. The 1968 nm absorption can provide insights into phenolic content, oxidation status, and potential degradation due to thermal processing or prolonged storage. Additionally, it helps differentiate natural phenolic profiles across various EVOO varieties and can be used to assess adulteration or dilution with lower-quality oils. Comparing 1968 nm spectral data with reference EVOO spectra enables quality control, authenticity verification, and monitoring of phenolic stability under different processing and storage conditions.

The SELECT procedure efficiently narrowed down the relevant variables, selecting only a subset of wavelengths from the full NIR spectral range. The final model, which uses just these 15 wavelengths, has been optimized for prediction while avoiding overfitting and unnecessary complexity. This procedure provides a more streamlined model with better predictive performance than using the full 700-variable NIR dataset. By focusing on a relatively small number of key wavelengths, the model becomes both efficient and effective for predicting caffeic acid content in various olive oil categories. This method allows rapid, non-destructive analysis of olive oil quality and can be easily implemented in quality control and certification processes within the olive oil industry.

Secoiridoids, though rare in most plants, are abundant in *Olea europaea* leaves and fruits. However, due to their oil insolubility, only a small portion transfer to EVOO during extraction [54]. Despite this, they are key micronutrients, contributing to EVOO’s sensory properties and health benefits. The most common secoiridoids in EVOO include oleuropein and ligstroside aglycones [7,54,55].

The SELECT approach identified 30 significant wavelengths out of 700 for quantifying decarboxymethyl ligstroside aglycone in dialdehyde form (oleocanthal) (Table 8), with the 2084 nm having the first order of selection, followed by 1220 nm. In the first order of selection, the results revealed that lower-quality olive oils, such as pomace olive oil, olive oil with °1 and °0.4 acidity, as well as Hojiblanca, exhibited higher absorbance at 2084 nm, indicating increased oxidation and lower stability compared with EVOO varieties like Manzanilla, Picual, Koroneiki, Arbosana, and Royuela. This suggests that EVOOs may differ in stability due to their higher phenolic content, which contributes to both oxidative resistance and unique sensorial attributes, as reported earlier by Mehany et al. [40].

The wavelength around 2084 nm is also associated with the –COOR and C–H stretching vibrations, along with C=O stretching, which are sensitive to oxidative changes and degradation in olive oil [20,46]. This further reinforces the role of 2084 nm in detecting oxidative stress and quality degradation, which is particularly relevant when monitoring the stability of olive oils under varying processing conditions.

By incorporating this wavelength into the chemometric model, the SELECT-OLS method enables reliable prediction of oleocanthal in various olive oil types, providing an efficient and accurate way to monitor this compound under different processing conditions, such as supplemented and non-supplemented oils and deep-fried versus non-fried samples. This demonstrates the potential of NIR spectroscopy combined with variable selection for quantifying specific phenolic compounds like oleocanthal in olive oils.

Moreover, the current results demonstrate that the SELECT approach successfully identified 30 spectral markers out of 700 variables for quantifying oleacein content in various olive oil categories (Table 9). The first-order selection was at 2006 nm, followed by 2216 nm. The results indicated that lower-quality olive oils, such as pomace olive oil, olive oils with °1 and °0.4, and Hojiblanca, exhibited higher absorbance at 2006 nm, suggesting increased oxidation and lower stability compared with EVOO varieties like Cornicabra, Picual, Manzanilla, Arbequina, and Royuela. These findings imply that EVOOs may vary in stability due to their higher phenolic content, which contributes to both oxidative resistance and distinctive sensorial attributes. Indeed, oleacein, a key secoiridoid compound in EVOO, has been shown to enhance mitochondrial function by increasing mitochondrial mass, DNA content, respiration, and ATP production in colorectal cancer cells. It triggers a protective cellular response involving antioxidant pathways mediated by AMPK, NRF2, and PGC-1α. Oleacein acts as a partial agonist of PPARγ, a receptor involved in regulating mitochondrial metabolism. Its beneficial effects on mitochondrial pathways and antioxidant defense are significantly mediated through PPARγ activation [56].

The wavelength around 2006 nm is associated with –COOR and C–H stretching vibrations, as well as C=O stretching, which are sensitive to oxidative changes and degradation in olive oil. Additionally, the same trend of high oxidation was observed in oil samples fried at a high temperature (210 °C) for an extended time (6 h). This reinforces the role of 2006 nm in detecting oxidative stress and quality degradation, which is particularly relevant for monitoring the stability of olive oils under different processing conditions.

**Figure 5:**
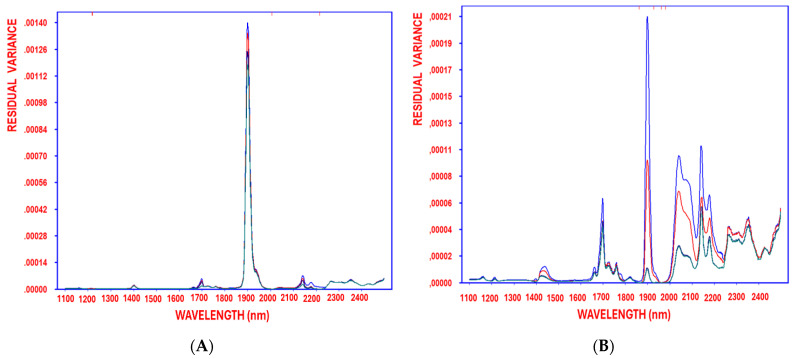

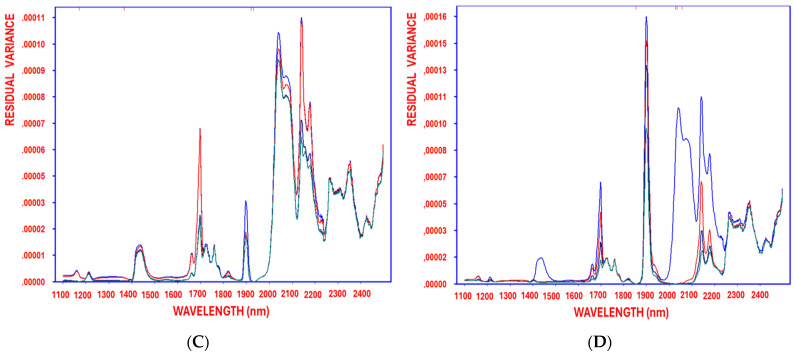
Residual variance of the variables decorrelated by SELECT after 1, 2, 3, and 4 (blue, red, turquoise, green, respectively) selections obtained when working on auto-scaled NIR spectra, using oleacein (**A**), homovanillic acid (**B**), pinoresinol (**C**), and OAOAH (**D**) as response variables.

Moreover, the continuous reduction in residual variance, observed from the initial stage before variable selection through each decorrelation cycle until the optimal model complexity was reached, further validates the method’s effectiveness in minimizing residual variance (Figure 5A–D), using oleacein, homovanillic acid, pinoresinol, and OAOAH as response variables, respectively.

Additionally, SELECT identified 21 key variables out of 700 wavelengths for quantifying homovanillic acid across various olive oil categories (Table 10). Among these, the 1962 nm wavelength had the first order of selection, followed by 1862 nm. The 1962 nm wavelength in NIR is significant due to its association with the O–H (hydroxyl) group and C=O (carbonyl) stretching vibrations, which are sensitive to secondary oxidation products such as aldehydes and ketones. These functional groups are key indicators of oxidative degradation in olive oil, and their presence can be used for assessing the oil’s quality [45]. Thus, the 1962 nm wavelength plays a critical role in monitoring secondary oxidation, especially in detecting aldehydes and ketones that form during the oxidative process. These oxidation products contribute to the rancidity and degradation of olive oils, making this wavelength valuable for quality control.

The 1962 nm wavelength showed strong absorbance, especially in HTyr-supplemented and non-fried EVOO, making it effective for distinguishing between pure and supplemented oils. Variations in absorbance at this wavelength can indicate dilution effects from mixing non-virgin oils with fruit extract, which may affect quality. Comparing absorption intensities across pure, supplemented, fried, and non-fried samples confirmed the utility of NIR spectroscopy combined with SELECT for differentiating treatment conditions. This wavelength is also sensitive to oxidation products, aiding in the detection of oxidative degradation. Most EVOO varieties, including Picual, Cornicabra, Koroneiki, Royuela, Arbequina, and Manzanilla, showed lower oxidation values, reflecting higher stability that is likely to have been due to their rich phenolic profiles and distinct sensory characteristics [40].

Regarding pinoresinol, the SELECT method identified 30 key wavelengths out of 700 for its quantification under varying supplementation and frying conditions. As detailed in Table 11, the most significant wavelengths were 1932 nm and 1922 nm. The 1932 nm wavelength played a dominant role, presumably due to its sensitivity to molecular vibrations associated with phenolic compounds like pinoresinol. The 1922 nm wavelength also contributed by capturing complementary spectral features, refining the model’s accuracy. These wavelengths, along with the others selected, form the foundation of the SELECT-OLS model for pinoresinol prediction, demonstrating high accuracy with minimal input variables. The method’s efficiency is further validated by the consistent decline in residual variance during variable selection and decorrelation cycles, confirming the model’s robustness and utility for precise olive oil quality assessment. In addition, our quantitative analysis (Table 3) confirmed that pinoresinol content was significantly higher in the EVOO compared with the refined and blended olive oils. For instance, pomace (orujo) oil contained no detectable pinoresinol, while olive oil 0.4° showed 2.58 mg/kg. These results align with previous findings by Cecchi et al. [57], who reported that pinoresinol is relatively stable and less susceptible to degradation during the refining process.

From the 700 NIR-recorded variables, the SELECT method identified 30 key wavelengths for quantifying OAOAH content in olive oils under different supplementation and frying conditions (Table 12). The most critical wavelength was 1854 nm, followed by 2028 nm. The 1854 nm wavelength is particularly significant, reflecting molecular interactions linked to oxidative degradation and phenolic content. Its high absorbance in the HTyr-supplemented oils suggests that it detected externally added antioxidants rather than those intrinsic to EVOO. This confirms the capability of NIR combined with SELECT to differentiate between supplemented and non-supplemented oils. The efficiency of this approach is further supported by the continuous reduction in residual variance through the decorrelation cycle. The SELECT-OLS model, using only the most relevant variables, delivers high predictive accuracy while remaining cost-effective and suitable for routine quality assessment. This method is especially valuable for predicting OAOAH levels across extra virgin, virgin, and refined olive oils, serving as a reliable marker of oxidative stability and the oil’s authenticity. Its rapid, non-destructive nature supports online and in-process quality control, with minimal preparation and high accuracy.

**Figure 6:**
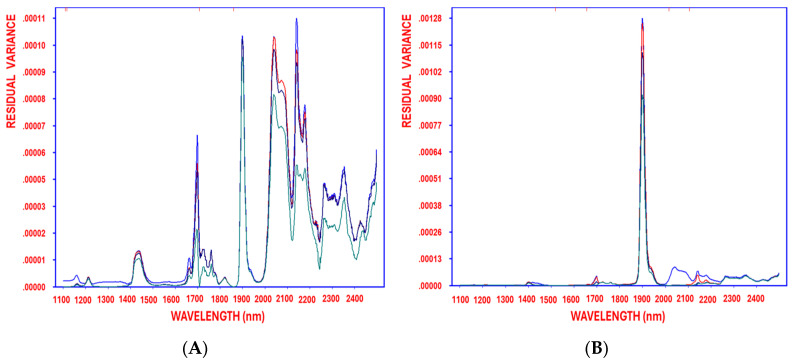
Residual variance of the variables decorrelated by SELECT after 1, 2, 3, and 4 (blue, red, turquoise, green, respectively) selections obtained when working on auto-scaled NIR spectra, using LAOAH (**A**) and TPC (**B**) as response variables.

Furthermore, from the 700 variables recorded in the NIR system, the SELECT method identified 30 key variables for quantifying LAOAH in olive oils under different supplementation and frying conditions (Table 13). The results showed that 1860 nm was the most significant wavelength, followed by 1110 nm. Particularly high absorbance at 1860 nm was observed in olive oils supplemented with HTyr, suggesting that the extract was added during processing rather than being part of the original EVOO content. This underscores NIR and SELECT’s ability to differentiate between oils supplemented with external antioxidants and those that are not. The method’s efficiency is further validated by the continuous reduction in residual variance throughout the decorrelation cycle. This consistent decrease, observed from the initial stage before variable selection through each successive cycle until optimal model complexity was achieved, demonstrates the method’s effectiveness in minimizing residual variance (Figure 6A,B), using LAOAH and TPC as response variables, respectively.

In the quantification of total phenolic content, the SELECT method identified 18 key wavelengths from the 700 NIR-recorded variables across various olive oil supplementation and frying conditions (Table 14). The most significant wavelength was 1518 nm, followed by 2016 nm. The 1518 nm band is linked to C-H stretching vibrations in lipids and water and is commonly used in food oil analysis. In olive oil, this wavelength provides information about fatty acid composition and oxidative degradation, particularly the presence of oxidized lipids such as aldehydes and ketones. It is also sensitive to hydrogen bonding and can detect changes due to storage or heat exposure. High absorbance at 1518 nm was observed in HTyr-supplemented oils, reflecting their elevated phenolic content and allowing clear differentiation from pure EVOO. This highlights the effectiveness of this wavelength in identifying supplementation effects and oxidative changes. Moreover, pomace olive oil and Hojiblanca, when fried at high temperatures for extended durations, showed the highest levels of aldehydes and ketones, confirming their lower oxidative stability. In contrast, EVOOs, especially those with HTyr supplementation, maintained better stability due to their natural antioxidant content.

### 3.4. SELECT-OLS Models Validation Assessment

The statistical characteristics of the developed NIR and SELCT-OLS models for quantifying phenolic compounds in various olive oils, including HTyr supplementation and deep frying, are illustrated in Table 15. The standard deviation of the error (SDE) reflects the average deviation between observed and predicted values in the model, with a lower SDE indicating better model fit and more accurate predictions. For instance, hydroxytyrosol has an SDE of 19.51, attributed to the high HTyr content in the analyzed samples, particularly in olive oils supplemented with olive fruit extract, a rich source of HTyr and its derivatives. In contrast, compounds like homovanillic acid show better precision with an SDE of 3.89. The mean absolute error (MAE), which measures the average magnitude of prediction errors, similarly indicates better accuracy when smaller. Caffeic acid, with the smallest MAE of 3.19, demonstrated more accurate predictions compared with compounds like LAOAH (MAE = 69.87). This difference is likely to have been due to variations in the content of phenolic compounds and their dynamics under deep-frying conditions. The multiple correlation coefficient (R) assesses the strength and direction of the linear relationship between observed and predicted values. An R value close to 1 indicates a strong correlation; the results for most of the compounds, including hydroxytyrosol, tyrosol, and caffeic acid (R = 0.98), demonstrated excellent prediction accuracy. Pinoresinol, with a slightly lower R value of 0.91, still demonstrated a good prediction model. Leave-one-out residual variance (LOORV) measured the model’s stability when one data point was left out at a time, with lower values indicating better generalizability. Caffeic acid had the lowest LOORV of 4.57%, indicating high stability. Similarly, the leave-one-out residual standard deviation (LOORSD), measured in standard deviation units, for caffeic acid was relatively low at 4.87%, suggesting consistent predictions. The leave-one-out explained variance (LOOEV) shows how much of the data’s total variance the model explains, with most compounds showing high values (above 79%) and hydroxytyrosol reaching 95%. The leave-one-out mean prediction error (LOOMPE) measured the average error when one data point was excluded, with caffeic acid again performing best with a LOOMPE of 3.64. In conclusion, most phenolic compounds were predicted excellently by the models, as evidenced by their high correlation coefficients (R = 0.91–0.98) and low prediction errors. These compounds were quantified with high precision and low residual variance, making the models ideal for reliable quantification of olive oil. Total phenolic content (TPC), with an R value of 0.96, showed a high explained variance (90.14%) and low mean prediction error, suggesting a strong and accurate model. Overall, the models for quantifying phenolic compounds in olive oils are robust, particularly for hydroxytyrosol, tyrosol, and caffeic acid, for which these models are highly reliable.

Overall, the results demonstrate the robustness and flexibility of SELECT-OLS as an effective feature selection and correction method for quantifying phenolic content across various olive oil types, including both fried and non-fried oils, and those supplemented with exogenous phenolic compounds or without supplementation. By systematically reducing dimensionality while preserving high predictive performance, SELECT-OLS optimizes model complexity, minimizes residual variance, and improves the accuracy of calibration models for phenolic substances in complex oil matrices. The consistency observed across different phenolic compounds further highlights the reliability of SELECT-OLS for spectral data analysis and quantitative modeling.

The goal of this study was achieved by developing regression models to quantify the phenolic content of different olive oil samples based on measured spectral data. In SELECT-OLS, predictions are made by transforming inter-correlated variables into a set of independent factors, known as latent variables (LVs), which capture the maximum covariance between spectral data and response variables such as HTyr, Tyr, caffeic acid, oleocanthal, oleacein, homovanillic acid, pinoresinol, OAOAH, LAOAH, and TPC. Each SELECT-OLS model’s LVs are statistically independent (uncorrelated) and contain all relevant information necessary for stable predictions. As noted in recent studies, only the first few LVs account for the majority of variation in the original variables, while the remaining LVs primarily represent random noise or linear dependencies [58,59]. This also aids in understanding multivariate data analytics algorithms [60,61].

## 4. Conclusions

In the current study, NIR spectroscopy combined with selection of variables using SELECT and OLS regression proved to be an effective approach for developing robust, reduced-spectrum regression models to quantify key phenolic compounds in various olive oils. These models successfully identified and quantified compounds such as HTyr, Tyr, caffeic acid, oleocanthal, oleacein, homovanillic acid, pinoresinol, OAOAH, LAOAH, and TPC across different olive oil types, both supplemented and non-supplemented with HTyr, before and after deep frying under various thermal stress conditions. The SELECT-OLS models demonstrated high predictive accuracy, with correlation coefficients (R) ranging from 0.91 to 0.98, using at most 30 wavelengths from a total of 700, ensuring minimal model complexity. These models performed reliably across diverse phenolic supplementation and deep-frying conditions, offering dependable tools for routine, online prediction and quantification of phenolic compounds. Furthermore, the optimized variable selection in NIR spectroscopy simplifies spectral analysis, reduces costs, and highlights the feasibility of NIR as a rapid, non-destructive, and eco-friendly tool for evaluating and quantifying edible oils.

## Figures and Tables

**Table 1:** Experimental design (2^3^ factorial) of several olive oil types supplemented with olive fruit extract under various deep-frying conditions, including 8 treatments and 3 controls before frying.

Experiment	Design Matrix			Independent Variables			Response
	X_1_	X_2_	X_3_	Time (h)	Temp. (°C)	Polyphenols (mg/kg)	
1	−1	−1	−1	3	170	-	Phenolic compounds
2	+1	−1	−1	6	170	-	
3	−1	+1	−1	3	210	-	
4	+1	+1	−1	6	210	-	
5	−1	−1	+1	3	170	650	
6	+1	−1	+1	6	170	650	
7	−1	+1	+1	3	210	650	
8	+1	+1	+1	6	210	650	
Level	Time (h)	Temp. (°C)	Polyphenols (mg/kg)				
−1	3	170	Original concentration (0 addition)				
+1	6	210	650				

**Table 2:** Representative sampling design of various olive oil categories used in this study.

Olive Oil Type	Cultivar/Category	Type/Blend	Source/Brand	Location	Notes
Extra Virgin Olive Oil (EVOO)	Picual	Single variety EVOO	La casa del aceite, S.L. Cascante, Navarra	Spain	Spanish variety
EVOO	Cornicabra	Single variety EVOO	Aceite del Sur, COOSUR, Vilches, Jaén	Spain	Spanish variety
EVOO	Empeltre	Single variety EVOO	La casa del aceite, Navarra	Spain	Spanish variety
EVOO	Arbequina	Single variety EVOO	Aceite del Sur, COOSUR, Vilches, Jaén	Spain	Spanish variety
EVOO	Hojiblanca	Single variety EVOO	Aceite del Sur, COOSUR, Vilches, Jaén	Spain	Spanish variety
EVOO	Manzanilla Cacereña	Single variety EVOO	Aceite Artajo, Finca Los Llanos s/n, Fontellas, Navarra	Spain	Spanish variety
EVOO	Royuela/Arróniz	Single variety EVOO	Aceite Artajo, Finca Los Llanos s/n, Fontellas, Navarra	Spain	Spanish variety
EVOO	Koroneiki	Single variety EVOO	Aceite Artajo, Finca Los Llanos s/n, Fontellas, Navarra	Spain	Greek-origin variety cultivated in Spain
EVOO	Arbosana	Single variety EVOO	Aceite Artajo, Finca Los Llanos s/n, Fontellas, Navarra	Spain	Spanish variety
EVOO and refined olive oil	Olive oil 1%	Blend	La Masia, Oleo Masia, S.A. Sevilla	Spain	Spanish variety
Refined olive oil and virgin olive oil (VOO)	Olive oil 0.4%	Blend	La Española oils, Seville	Spain	Spanish variety
Orujo olive oil (pomace) and EVOO	Orujo	Blend	Simply	Spain	Spanish variety

**Table 3:** Evolution and changes in phenolic compounds in olive oils as affected by cultivar, hydroxytyrosol supplementation, and deep-frying. Data are presented as the mean of three replicates, prior to use in regression models.

Samples	HTyr	Tyr	Caffeic Acid	Oleocanthal	Oleacein	Homovanillic Acid	Pinoresinol	OAOAH	LAOAH	TPC
PC_C1	11.89	7.51	0.00	31.12	73.26	0.00	19.87	4.14	6.97	307.89
PC_S	359.91	195.59	90.99	110.36	33.99	46.92	29.82	7.46	23.73	1524.32
PC_C2	125.28	70.45	46.19	14.44	54.77	18.74	44.53	4.92	6.47	658.60
PC_1	9.89	6.62	0.00	45.71	131.46	0.00	15.78	65.42	184.37	589.94
PC_2	9.54	5.31	0.00	41.97	105.29	0.00	22.54	94.53	347.34	819.73
PC_3	10.66	6.44	0.00	53.31	130.41	0.00	17.05	36.72	185.68	537.89
PC_4	2.65	5.40	0.00	37.94	38.61	0.00	16.91	51.60	356.54	696.80
PC_5	124.50	71.86	42.03	63.78	153.10	18.08	48.00	78.34	223.28	1107.15
PC_6	102.51	59.73	29.96	59.32	124.09	15.33	46.59	96.99	329.23	1159.89
PC_7	120.01	72.07	34.20	39.88	151.41	16.25	34.14	24.56	207.78	969.29
PC_8	62.78	47.00	27.86	28.04	93.04	6.69	20.43	32.05	298.56	944.61
CC_C1	18.13	12.00	0.00	34.91	31.91	0.00	17.36	18.16	7.25	275.67
CC_S	389.49	215.50	83.36	62.49	153.37	60.21	71.30	7.36	5.58	1683.03
CC_C2	128.57	79.63	36.15	28.56	24.97	21.39	51.24	6.14	11.31	658.14
CC_1	17.11	11.96	0.00	88.28	77.60	0.00	15.49	128.09	360.85	847.25
CC_2	10.95	10.28	0.00	54.77	59.65	0.00	13.99	125.54	399.34	836.85
CC_3	13.53	10.49	0.00	60.60	88.96	0.00	13.73	38.06	276.31	588.02
CC_4	8.85	8.83	0.00	51.28	62.95	0.00	16.17	35.85	347.69	679.57
CC_5	122.86	80.12	33.33	52.73	44.84	17.63	48.04	149.29	411.57	1276.62
CC_6	79.19	67.76	31.02	45.26	26.93	15.87	50.14	149.04	616.28	1376.11
CC_7	115.76	73.58	29.10	66.27	74.43	17.12	50.04	70.08	325.43	997.90
CC_8	76.33	47.69	30.31	61.43	67.47	11.91	45.44	68.69	462.20	1126.87
EP_C1	17.80	7.99	0.00	42.11	79.23	0.00	9.45	25.20	5.81	337.91
EP_S	363.50	193.04	69.57	22.50	140.58	21.02	47.95	15.20	12.81	1468.88
EP_C2	122.44	65.08	24.97	36.97	73.83	15.82	43.95	11.73	12.01	647.23
EP_1	16.65	7.56	0.00	57.43	136.38	0.00	10.66	96.76	304.95	821.61
EP_2	11.55	6.92	0.00	58.54	120.45	0.00	13.79	92.61	368.61	895.81
EP_3	10.85	7.90	0.00	64.69	118.54	0.00	7.27	53.27	330.76	775.75
EP_4	9.85	7.00	0.00	56.86	84.57	0.00	5.66	48.93	339.26	741.32
EP_5	91.14	56.90	22.77	53.05	115.58	13.96	41.45	91.75	303.27	1062.90
EP_6	82.53	54.57	21.17	56.88	116.60	11.42	43.55	89.95	346.85	1104.52
EP_7	64.44	41.78	17.36	65.79	116.95	5.65	32.13	35.58	276.04	858.28
EP_8	50.59	34.93	15.11	62.38	97.24	4.83	27.83	28.90	226.27	734.49
AQ_C1	8.84	9.56	0.00	24.44	24.26	0.00	34.22	3.62	7.37	227.71
AQ_S	332.69	177.98	62.58	17.81	14.65	20.74	41.94	13.46	4.51	1384.96
AQ_C2	139.29	89.17	32.32	15.05	14.91	18.27	57.75	11.12	11.53	663.96
AQ_1	5.46	7.36	0.00	34.74	33.39	0.00	25.62	138.03	513.57	940.33
AQ_2	2.59	6.55	0.00	34.31	31.96	0.00	27.82	118.61	534.12	945.26
AQ_3	5.56	6.84	0.00	45.07	43.42	0.00	29.16	74.61	428.93	781.58
AQ_4	4.38	6.79	0.00	43.08	40.60	0.00	29.22	63.83	400.00	715.60
AQ_5	122.19	87.12	35.78	25.65	16.01	21.06	54.29	144.73	456.38	1279.66
AQ_6	96.45	77.45	28.80	26.35	18.33	17.41	54.92	176.65	686.59	1534.98
AQ_7	99.99	71.26	28.47	30.67	20.34	15.08	52.04	134.05	559.03	1335.45
AQ_8	65.33	51.57	26.63	31.72	28.58	9.44	46.77	73.09	514.76	1133.19
HB_C1	13.53	14.86	0.00	22.83	15.72	0.00	20.68	5.46	8.55	209.51
HB_S	320.65	174.49	68.55	4.06	19.99	44.46	35.34	10.86	4.34	1208.67
HB_C2	186.03	98.67	32.57	18.95	10.30	17.30	51.49	8.16	9.35	655.73
HB_1	9.05	13.88	0.00	31.71	17.62	0.00	19.88	107.68	278.19	598.94
HB_2	2.73	12.66	0.00	24.55	9.49	0.00	22.13	116.35	405.57	807.24
HB_3	9.79	12.49	0.00	38.23	32.85	0.00	19.50	28.48	178.72	377.85
HB_4	2.64	9.74	0.00	33.52	16.97	0.00	19.99	40.38	275.02	553.77
HB_5	132.31	81.90	30.05	27.98	24.16	17.12	48.16	105.33	312.31	1044.73
HB_6	92.97	66.19	27.62	24.35	17.80	19.00	42.70	107.12	400.14	1093.86
HB_7	112.27	63.62	31.83	31.24	26.74	18.88	45.66	14.38	106.19	639.49
HB_8	65.10	39.61	26.99	30.96	27.03	14.47	38.81	21.46	154.96	637.05
MZ_C1	7.28	9.36	0.95	29.01	35.06	0.00	50.22	3.66	7.64	309.04
MZ_S	347.51	181.16	70.08	29.40	22.74	39.62	109.11	24.79	14.46	1255.28
MZ_C2	156.67	78.04	29.93	17.22	17.20	19.17	50.83	14.10	10.06	661.28
MZ_1	4.44	8.93	0.00	66.60	85.93	0.00	21.27	40.23	125.85	428.46
MZ_2	4.13	8.45	0.00	62.23	64.93	0.00	22.89	51.60	216.92	604.60
MZ_3	4.54	11.10	0.00	72.96	73.23	0.00	20.97	6.67	43.47	255.57
MZ_4	2.71	9.96	0.00	62.41	38.28	0.00	19.35	11.98	92.00	338.04
MZ_5	128.99	79.33	30.79	56.96	59.05	17.22	52.00	43.72	147.92	870.67
MZ_6	117.15	76.65	28.41	45.40	50.65	15.80	48.74	54.22	206.21	945.18
MZ_7	138.43	79.42	28.94	73.23	81.42	13.19	49.61	9.78	71.34	723.96
MZ_8	90.05	57.34	27.52	59.94	55.30	8.61	42.76	19.05	125.95	727.40
RY_C1	21.43	5.87	0.00	23.45	116.26	0.00	65.19	6.30	21.80	400.63
RY_S	290.17	166.02	67.77	26.93	30.43	47.86	37.60	11.09	7.43	1211.16
RY_C2	120.00	62.49	26.38	22.69	61.15	13.15	42.38	13.21	9.22	662.38
RY_1	20.05	5.88	0.00	57.46	199.34	0.00	22.13	38.34	126.15	539.16
RY_2	15.43	5.17	0.00	47.22	132.34	0.00	22.18	64.32	274.21	691.69
RY_3	15.63	6.03	0.00	55.55	156.57	0.00	21.46	15.28	100.20	415.14
RY_4	11.66	5.24	0.00	49.89	99.76	0.00	20.65	17.26	144.43	428.41
RY_5	110.14	62.04	22.78	51.90	166.92	12.05	40.76	38.73	145.25	854.57
RY_6	90.65	52.72	20.45	46.13	134.40	11.18	39.19	57.27	256.38	937.29
RY_7	97.86	58.31	21.73	50.87	149.07	14.32	35.31	11.52	79.56	688.53
RY_8	72.80	44.52	18.88	49.58	125.45	9.89	30.88	20.75	146.43	688.71
OJ_C1	0.00	0.00	0.00	0.00	2.65	0.00	0.00	0.00	0.00	3.89
OJ_S	338.49	197.82	86.16	112.01	6.22	52.80	48.45	5.39	5.60	1259.06
OJ_C2	190.09	95.18	48.26	49.30	11.16	21.09	53.06	0.00	0.00	652.25
OJ_1	0.00	0.00	0.00	0.00	0.00	0.00	0.00	7.48	25.56	49.11
OJ_2	0.00	0.00	0.00	0.00	0.00	0.00	0.00	37.11	168.70	298.40
OJ_3	0.00	0.00	0.00	0.00	0.00	0.00	0.00	12.72	75.98	119.62
OJ_4	0.00	0.00	0.00	0.00	0.00	0.00	0.00	44.40	281.70	426.64
OJ_5	163.00	83.88	39.68	8.26	0.00	17.97	50.05	15.62	71.60	657.61
OJ_6	140.73	76.71	39.44	6.44	0.00	16.83	50.89	36.04	175.42	803.54
OJ_7	130.51	64.62	36.44	26.04	0.00	13.61	49.75	9.14	65.47	559.38
OJ_8	84.60	44.32	32.40	5.70	0.00	15.77	41.53	25.67	167.59	609.17
KN_C1	15.14	12.66	0.00	40.50	45.18	0.00	68.80	0.00	0.00	327.77
KN_S	275.46	154.99	68.30	37.99	19.49	49.22	48.80	37.16	0.00	1230.89
KN_C2	113.74	65.86	25.71	20.08	34.30	16.94	44.64	8.11	12.32	663.93
KN_1	11.62	8.92	0.00	10.86	113.93	0.00	39.84	135.19	29.87	564.96
KN_2	10.20	8.76	0.00	73.01	87.37	0.00	11.38	54.92	202.13	642.58
KN_3	11.96	9.08	0.00	85.25	107.27	0.00	8.25	25.65	134.52	473.15
KN_4	8.71	8.15	0.00	76.68	60.39	0.00	8.84	28.05	217.57	588.67
KN_5	111.37	65.18	25.56	73.56	98.76	16.83	46.74	46.71	127.38	876.73
KN_6	101.32	63.74	26.80	72.67	94.17	15.01	48.39	61.08	208.02	986.50
KN_7	85.57	60.85	20.10	79.46	84.19	8.11	34.28	13.08	129.50	697.12
KN_8	53.14	41.26	0.00	69.45	64.50	3.59	28.73	16.60	162.42	662.45
AS_C1	13.87	11.26	0.00	47.74	57.62	0.00	56.91	17.29	5.24	393.00
AS_S	301.93	153.99	78.29	44.61	26.60	55.85	61.97	17.62	5.88	1434.58
AS_C2	97.65	54.60	20.57	51.98	30.39	12.59	46.82	15.02	5.41	666.95
AS_1	10.26	7.11	0.00	85.80	130.84	0.00	57.25	57.47	160.79	631.38
AS_2	10.77	7.82	0.00	82.86	112.85	0.00	59.19	63.76	238.83	749.95
AS_3	7.78	8.66	0.00	90.03	85.28	0.00	56.57	8.48	128.73	451.35
AS_4	7.65	7.02	0.00	75.21	61.66	0.00	51.26	12.60	159.54	486.19
AS_5	96.69	55.47	25.37	80.77	122.86	13.37	42.39	61.64	167.09	956.98
AS_6	89.06	55.29	21.55	76.57	105.99	11.56	44.98	68.30	248.22	1032.78
AS_7	84.46	56.94	17.93	85.03	111.77	12.10	34.99	36.97	182.61	818.89
AS_8	65.04	44.71	15.47	76.60	88.64	8.87	30.88	35.94	234.29	839.94
1ºO_C1	21.91	11.42	0.00	16.18	20.71	0.00	46.04	7.77	4.08	181.88
1ºO_S	283.72	148.32	63.35	15.44	16.58	41.06	102.13	18.95	10.61	1152.24
1ºO_C2	130.27	82.37	32.12	19.14	13.60	21.92	55.48	4.51	2.72	653.11
1ºO_1	19.23	11.51	0.00	28.20	27.16	0.00	36.74	87.19	239.75	568.77
1ºO_2	10.46	10.91	0.00	20.98	16.82	0.00	30.25	94.33	358.21	746.04
1ºO_3	14.08	10.14	0.00	30.04	38.05	0.00	15.11	46.75	227.41	454.87
1ºO_4	8.88	8.42	0.00	26.22	23.84	0.00	11.15	35.42	263.41	525.17
1ºO_5	121.42	73.01	27.83	26.14	24.25	16.60	47.80	93.18	258.61	968.57
1ºO_6	99.73	64.56	24.58	23.47	18.55	15.66	46.65	101.27	337.55	1057.58
1ºO_7	98.01	58.17	25.64	23.88	27.43	13.43	43.32	30.05	204.18	713.34
1ºO_8	62.03	37.70	21.11	24.78	25.89	9.51	39.22	18.54	181.50	623.20
0.4ºO_C1	0.00	0.00	0.00	4.95	4.05	0.00	2.58	0.00	0.00	26.50
0.4ºO_S	315.35	156.61	71.36	15.27	5.30	46.91	104.42	20.16	10.65	1113.40
0.4ºO_C2	180.75	97.66	42.93	9.48	4.82	26.84	63.12	0.00	0.00	650.87
0.4ºO_1	0.00	0.00	0.00	6.33	3.60	0.00	1.67	19.60	71.28	144.45
0.4ºO_2	0.00	0.00	0.00	6.26	2.06	0.00	1.52	45.41	206.83	392.82
0.4ºO_3	0.00	0.00	0.00	5.78	4.13	0.00	1.16	12.64	69.86	131.76
0.4ºO_4	0.00	0.00	0.00	5.86	2.36	0.00	1.10	17.02	176.36	333.24
0.4ºO_5	161.46	93.19	39.61	4.64	4.70	24.13	61.22	26.27	103.13	776.85
0.4ºO_6	120.07	76.56	34.76	4.11	3.99	19.70	53.41	49.82	225.78	886.43
0.4ºO_7	144.97	78.48	40.63	7.87	0.00	18.66	61.70	11.87	107.97	721.89
0.4ºO_8	76.32	45.70	31.45	6.41	0.00	9.66	49.00	20.41	175.28	650.79

PC_C1: Picual_Control 1; PC_S: Picual_Supplemented; PC_C2: Picual_Control 2; PC_1: Picual_Exp 1; PC_2: Picual_Exp 2; PC_3: Picual_Exp 3; PC_4: Picual_Exp 4; PC_5: Picual_Exp 5; PC_6: Picual_Exp 6; PC_7: Picual_Exp 7; PC_8: Picual_Exp 8; CC_C1: Cornicabra_Control 1; CC_S: Cornicabra_Supplemented; CC_C2: Cornicabra_Control 2; CC_1: Cornicabra_Exp 1; CC_2: Cornicabra_Exp 2; CC_3: Cornicabra_Exp 3; CC_4: Cornicabra_Exp 4; CC_5: Cornicabra_Exp 5; CC_6: Cornicabra_Exp 6; CC_7: Cornicabra_Exp 7; CC_8: Cornicabra_Exp 8; EP_C1: Empeltre_Control 1; EP_S: Empeltre_Supplemented; EP_C2: Empeltre_ Control 2; EP_1: Empeltre_Exp 1; EP_2: Empeltre_Exp 2; EP_3: Empeltre_Exp 3; EP_4: Empeltre_Exp 4; EP_5: Empeltre_Exp 5; EP_6: Empeltre_Exp 6; EP_7: Empeltre_Exp 7; EP_8: Empeltre_Exp 8; AQ_C1: Arbequina_Control 1; AQ_S: Arbequina_Supplemented; AQ_C2: Arbequina_ Control 2; AQ_1: Arbequina_Exp 1; AQ_2: Arbequina_Exp 2; AQ_3: Arbequina_Exp 3; AQ_4: Arbequina_Exp 4; AQ_5: Arbequina_Exp 5; AQ_6: Arbequina_Exp 6; AQ_7: Arbequina_Exp 7; AQ_8: Arbequina_Exp 8; HB_C1: Hojiblanca_Control 1; Hojiblanca_Supplemented; Hojiblanca_Control 2; HB_1: Hojiblanca_Exp 1; HB_2: Hojiblanca_Exp 2; HB_3: Hojiblanca_Exp 3; HB_4: Hojiblanca_Exp 4; HB_5: Hojiblanca_Exp 5; HB_6: Hojiblanca_Exp 6; HB_7: Hojiblanca_Exp 7; HB_8: Hojiblanca_Exp 8; MZ_C1: Manzanilla_ Control 1; MZ_S: Manzanilla_Supplemented; MZ_C2: Manzanilla_Control 2; MZ_1: Manzanilla_Exp 1; MZ_2: Manzanilla_Exp 2; MZ_3: Manzanilla_Exp 3; MZ_4: Manzanilla_Exp 4; MZ_5: Manzanilla_Exp 5; MZ_6: Manzanilla_Exp 6; MZ_7: Manzanilla_Exp 7; MZ_8: Manzanilla_Exp 8; RY_C1: Royuela_ Control 1; RY_S: Royuela_Supplemented; RY_C2: Royuela_ Control 2; RY_1: Royuela_Exp 1; RY_2: Royuela_Exp 2; RY_3: Royuela_Exp 3; RY_4: Royuela_Exp 4; RY_5: Royuela_Exp 5; RY_6: Royuela_Exp 6; RY_7: Royuela_Exp 7; RY_8: Royuela_Exp 8; OJ_C1: Pomace_Control 1; OJ_S: Pomace_ Supplemented; OJ_C2: Pomace_ Control 2; OJ_1: Pomace_Exp 1; OJ_2: Pomace_Exp 2; OJ_3: Pomace_Exp 3; OJ_4: Pomace_Exp 4; OJ_5: Pomace_Exp 5; OJ_6: Pomace_Exp 6; OJ_7: Pomace_Exp 7; OJ_8: Pomace_Exp 8; KN_C1: Koroneiki_Control 1; KN_S: Koroneiki_Supplemented; KN_C2: Koroneiki_Control 2; KN_1: Koroneiki_Exp 1; KN_2: Koroneiki_Exp 2; KN_3: Koroneiki_Exp 3; KN_4: Koroneiki_Exp 4; KN_5: Koroneiki_Exp 5; KN_6: Koroneiki_Exp 6; KN_7: Koroneiki_Exp 7; KN_8: Koroneiki_Exp 8; AS_C1: Arbosana_Control 1; AS_S: Arbosana_Supplemented; AS_C2: Arbosana_Control 2; AS_1: Arbosana_Exp 1; AS_2: Arbosana_Exp 2; AS_3: Arbosana_Exp 3; AS_4: Arbosana_Exp 4; AS_5: Arbosana_Exp 5; AS_6: Arbosana_Exp 6; AS_7: Arbosana_Exp 7; AS_8: Arbosana_Exp 8; 1°O_C1: Olive 1°_ Control 1; 1°O_S: Olive 1°_Supplemented; 1°O_C2: Olive 1°_ Control 2; 1°O_1: Olive 1°_Exp 1; 1°O_2: Olive 1°_Exp 2; 1°O_3: Olive 1°_Exp 3; 1°O_4: Olive 1°_Exp 4; 1°O_5: Olive 1°_Exp 5; 1°O_6: Olive 1°_Exp 6; 1°O_7: Olive 1°_Exp 7; 1°O_8: Olive 1°_Exp 8; 0.4°O_C1: Olive 0.4°_ Control 1; 0.4°O_S: Olive 0.4°_Supplemented; 0.4°O_C2: Olive 0.4°_ Control 2; 0.4°O_1: Olive 0.4°_Exp 1; 0.4°O_2: Olive 0.4°_Exp 2; 0.4°O_3: Olive 0.4°_Exp 3; 0.4°O_4: Olive 0.4°_Exp 4; 0.4°O_5: Olive 0.4°_Exp 5; 0.4°O_6: Olive 0.4°_Exp 6; 0.4°O_7: Olive 0.4°_Exp 7; 0.4°O_8: Olive 0.4°_Exp 8. Where, Control 1 (used as the control for Experiments 1–4) refers to original, non-deep-fried olive oil. Supplemented oil refers to non-deep-fried olive oil that had been enriched with olive fruit extract, which was also used in the preparation of Control 2. Control 2 (used as the control for Experiments 5–8) was a mixture of Control 1 and the supplemented oil, resulting in a total polyphenol content of up to 650 mg/kg. Exp.1 was olive oil deep-fried at 170 °C for 3 h without polyphenol supplementation, Exp.2 was olive oil deep-fried at 170 °C for 6 h without polyphenol supplementation, Exp.3 was olive oil deep-fried at 210 °C for 3 h without polyphenol supplementation, Exp.4 was olive oil deep-fried at 210 °C for 6 h without polyphenol supplementation, Exp.5 was olive oil deep-fried at 170 °C for 3 h with polyphenol supplementation, Exp.6 was olive oil deep-fried at 170 °C for 6 h with polyphenol supplementation, Exp.7 was olive oil deep-fried at 210 °C for 3 h with polyphenol supplementation, and Exp.8 was olive oil deep-fried at 210 °C for 6 h with polyphenol supplementation. Where, OAOAH: oleuropein aglycone, oxidized aldehyde and hydroxylic form; LAOAH: ligstroside aglycone, oxidized aldehyde and hydroxylic form; TPC: total phenolic content.

**Table 4:** Wavelength range (nm), functional groups, associated compounds, and their significance in olive oil analysis and oxidation detection.

Wavelength (nm)	Functional Groups	Assignment	Significance in Oil Analysis and Oxidation Detection	Source
1100–1150	–CH_3_	C–H stretching in lipids	Decetion of triglycerides and oil purity	[42,43,44,45]
1167	–CH_3_	C–H stretch 2nd overtone	Detection of triglycerides and oil purity	[20,46]
1208	–CH_2_	C–H stretch 2nd overtone	Free fatty acid estimation	[20,47]
1220	HC=CH–	C–H stretch 2nd overtone	Assessment of unsaturation levels, critical for nutritional value and oxidative stability	[48]
1392	–CH_3_	2C–H stretch + C–H deformation	Oils differentiation and fatty acid characterization	[20,46]
1414	–OH	O–H stretch	Oils differentiation and monitoring oil degradation	[20,49]
1724	–CH_2_, –CH_3_, =CH_2_	C–H 1st overtone	Detection of primary oxidation productsDegradation and oxidation assessment	[20,46]
1760	–CH_2_, –CH_3_, =CH_2_	C–H 1st overtone	Detection of primary oxidation productsDegradation and oxidation assessment	[50]
1900	O–H (Hydroxyl) group	O–H stretching	Deformation of hydroperoxides (ROOH), and assessing oxidative stability and quality control	[42,43,44,45]
1930–1950	O–H (Hydroxyl) group	C=O stretching	Secondary oxidation monitoring, assessing aldehydes and ketones, and useful for quality assessment of the oil	[42,43,44,45]
2022	–COOR	C–H stretch + C=O stretch	Detection of oxidation and rancidity and olive oil quality assessment	[20,46]
2049	–COOR	C–H stretch + C=O stretch	Detectionb of oxidative changes and degradation	[20,46]
2144	HC=CH–	C–H stretch + C=C stretch	Aldehydes and ketones formation from lipid degradation	[20,46]
2200–2300	C=O, –CH_2_–, and –CH_3_–	O–H and C–H combination bands	Hydrolysis and secondary oxidation assessment	[42,43,44,45]
2350–2500	–CH_2_– and –CH=CH–	C–H and O–H overtones	Detection of advanced lipid degradation and rancidity	[42,43,44,45]

**Table 5:** Chemometric details of the variable selection/decorrelation procedure carried out by SELECT, corresponding to the optimal OLS regression model developed from column auto-scaled NIR spectra. This model is proposed to quantify the hydroxytyrosol content of extra virgin, refined, and virgin olive oils.

Order of Selection	Predictor Index	Wavelength (nm)	Correlation Coefficient
1	432	1962	13,381.71346
2	379	1856	−19,674.36170
3	471	2040	−2295.36323
4	172	1442	33,590.38074
5	145	1388	−67,177.54010
6	64	1226	−30,164.76968
7	159	1416	−40,448.03140
8	193	1484	−40,430.36614
9	356	1810	−7959.20002
10	143	1384	48,504.25880
11	173	1444	121,733.59762
12	687	2472	1178.58109
Intercept	83.02092		

**Table 6:** Chemometric details of the variable selection/decorrelation procedure carried out by SELECT, corresponding to the optimal OLS regression model developed from column auto-scaled NIR spectra. This model is proposed to quantify the tyrosol content of extra virgin, refined, and virgin olive oils.

Order of Selection	Predictor (Original) Index	Wavelength (nm)	Correlation Coefficient
1	432	1962	6857.74742
2	379	1856	−11,765.70594
3	471	2040	−1448.50769
4	501	2100	18,388.42902
5	469	2036	147,906.20886
6	171	1440	12,127.19843
7	67	1232	−17,562.75333
8	162	1422	−33,122.62349
9	341	1780	2544.81899
10	182	1462	−11,396.59050
11	352	1802	−4174.90923
12	564	2226	−3601.86826
13	477	2052	−20,359.73093
14	166	1430	39,544.90655
Intercept	49.18834		

**Table 7:** Chemometric details of the variable selection/decorrelation procedure carried out by SELECT, corresponding to the optimal OLS regression model developed from column auto-scaled NIR spectra. This model is proposed to quantify the caffeic acid content of extra virgin olive oils, refined, and virgin olive oils.

Order of Selection	Predictor (Original) Index	Wavelength (nm)	Correlation Coefficient
1	435	1968	3835.45630
2	378	1854	−3831.74460
3	459	2016	−1019.82007
4	171	1440	7922.85146
5	146	1390	−20,396.29076
6	279	1656	−2858.14468
7	530	2158	2408.24660
8	543	2184	−3324.87634
9	372	1842	−6005.49065
10	161	1420	−6219.91960
11	167	1432	28,476.44287
12	386	1870	12,525.95903
13	366	1830	8481.03903
14	463	2024	−10,510.69378
15	535	2168	9185.45452
Intercept	19.66402		

**Table 8:** Chemometric details of the variable selection/decorrelation procedure carried out by SELECT, corresponding to the optimal OLS regression model developed from column auto-scaled NIR spectra. This model is proposed to predict the oleocanthal content of extra virgin, refined, and virgin olive oils.

Order of Selection	Predictor (Original) Index	Wavelength (nm)	Correlation Coefficient
1	493	2084	−973.10870
2	61	1220	3179.34659
3	60	1218	−35,914.37957
4	32	1162	−5062.88544
5	28	1154	25,979.19369
6	354	1806	−2969.11419
7	526	2150	2468.63699
8	122	1342	−16,987.85359
9	124	1346	60,171.73968
10	486	2070	12,653.54897
11	465	2028	−10,701.89750
12	499	2096	13,800.27621
13	507	2112	−13,875.11363
14	517	2132	8589.20041
15	502	2102	66,639.48526
16	62	1222	−53,120.18264
17	639	2376	757.05104
18	578	2254	−2359.59280
19	575	2248	2747.00328
20	602	2302	−2004.92531
21	570	2238	−3813.96484
22	646	2390	−1727.15655
23	637	2372	−3925.10362
24	595	2288	4414.91786
25	299	1696	−3080.57288
26	40	1178	18,876.78924
27	42	1182	−54,827.86908
28	572	2242	5307.74630
29	495	2088	−42,817.13491
30	520	2138	8912.73045
Intercept	40.60485		

**Table 9:** Chemometric details of the variable selection/decorrelation procedure carried out by SELECT, corresponding to the optimal OLS regression model developed from column auto-scaled NIR spectra. This model is proposed to quantify the oleacein content of extra virgin, refined, and virgin olive oils.

Order of Selection	Predictor (Original) Index	Wavelength (nm)	Correlation Coefficient
1	454	2006	−5680.06361
2	559	2216	4967.28419
3	61	1220	11,218.27851
4	60	1218	−67,297.61086
5	197	1492	−14,963.85598
6	311	1720	−1926.68819
7	322	1742	10,172.90761
8	172	1442	12,251.43432
9	200	1498	183,886.86250
10	549	2196	8976.99565
11	320	1738	−9690.73672
12	547	2192	−13,332.89685
13	323	1744	8936.87708
14	536	2170	7626.86144
15	340	1778	8774.22502
16	554	2206	38,724.02968
17	310	1718	13,885.03698
18	546	2190	−28,739.20861
19	59	1216	−86,830.86445
20	174	1446	−6,6198.23886
21	563	2224	−14,392.59184
22	568	2234	6843.62945
23	330	1758	−10,526.22840
24	319	1736	−10,684.44448
25	552	2202	52,941.22023
26	507	2112	7329.05989
27	211	1520	43,106.58213
28	177	1452	68,510.88996
29	514	2126	−10,193.90995
30	143	1384	10,586.05871
Intercept	56.93204		

**Table 10:** Chemometric details of the variable selection/decorrelation procedure carried out by SELECT, corresponding to the optimal OLS regression model developed from column auto-scaled NIR spectra. This model is proposed to quantify the homovanillic acid content of extra virgin, refined, and virgin olive oil.

Order of Selection	Predictor (Original) Index	Wavelength (nm)	Correlation Coefficient
1	432	1962	1803.82264
2	382	1862	−3185.60381
3	415	1928	1214.22297
4	441	1980	15,247.41441
5	342	1782	1293.83673
6	576	2250	−656.30091
7	343	1784	−9270.34062
8	509	2116	815.81331
9	298	1694	−1630.11451
10	289	1676	8402.71210
11	546	2190	−1198.27546
12	372	1842	−14,972.89837
13	49	1196	−3369.44202
14	530	2158	4207.78595
15	308	1714	−1218.14237
16	334	1766	1457.10606
17	431	1960	12,734.88053
18	346	1790	−4338.50419
19	491	2080	−516.84851
20	414	1926	10,753.56018
21	699	2496	232.21421
Intercept	10.76000		

**Table 11:** Chemometric details of the variable selection/decorrelation procedure carried out by SELECT, corresponding to the optimal OLS regression model developed from column auto-scaled NIR spectra. This model is proposed to quantify the pinoresinol content of extra virgin, refined, and virgin olive oils.

Order of Selection	Predictor (Original) Index	Wavelength (nm)	Correlation Coefficient
1	417	1932	940.26055
2	412	1922	−7229.33948
3	36	1170	−2222.22699
4	134	1366	6513.29833
5	368	1834	−2646.51335
6	350	1798	7312.16815
7	365	1828	10,722.64540
8	511	2120	798.70996
9	474	2046	−2723.24544
10	172	1442	13,752.73636
11	388	1874	10,525.32507
12	418	1934	−33,274.88057
13	567	2232	−4748.97864
14	652	2402	1195.20392
15	639	2376	−1197.03181
16	351	1800	−10,461.43040
17	510	2118	11,310.22065
18	359	1816	27,253.61862
19	425	1948	13,948.01071
20	569	2236	5891.40474
21	372	1842	16,657.38826
22	361	1820	−22,317.84085
23	343	1784	−3832.32465
24	39	1176	27,581.13642
25	346	1790	13,282.46818
26	38	1174	−50,538.23421
27	137	1372	−37,104.25827
28	386	1870	−31,285.16992
29	70	1238	−14,403.49116
30	71	1240	37,693.61572
Intercept	36.18394		

**Table 12:** Chemometric details of the variable selection/decorrelation procedure carried out by SELECT, corresponding to the optimal OLS regression model developed from column auto-scaled NIR spectra. This model is proposed to predict the OAOAH content of extra virgin, refined, and virgin olive oils.

Order of Selection	Predictor (Original) Index	Wavelength (nm)	Correlation Coefficient
1	378	1854	−7664.67224
2	465	2028	−778.56914
3	479	2056	17,208.69174
4	468	2034	−43,612.82562
5	511	2120	3675.37981
6	553	2204	−2352.47170
7	459	2016	−33,349.05465
8	473	2044	−45,325.72438
9	501	2100	−13,752.79497
10	478	2054	103,414.74005
11	451	2000	29,388.31732
12	294	1686	3802.10521
13	520	2138	−12,363.94991
14	142	1382	−9717.46283
15	173	1444	18,557.58366
16	565	2228	10,119.49859
17	542	2182	5334.01003
18	645	2388	−2111.64565
19	649	2396	4593.83527
20	461	2020	81,335.20840
21	523	2144	−10,767.74041
22	519	2136	15,190.96859
23	168	1434	34,017.42804
24	435	1968	−18,648.15455
25	40	1178	−27,541.37822
26	545	2188	27,163.66433
27	39	1176	84,555.71808
28	296	1690	−30,092.64793
29	546	2190	−26,058.31194
30	170	1438	−74,892.22478
Intercept	43.56524		

**Table 13:** Chemometric details of the variable selection/decorrelation procedure carried out by SELECT, corresponding to the optimal OLS regression model developed from column auto-scaled NIR spectra. This model is proposed to quantify the LOAOAH content of extra virgin, refined, and virgin olive oils.

Order of Selection	Predictor (Original) Index	Wavelength (nm)	Correlation Coefficient
1	381	1860	−35,235.15684
2	6	1110	21,773.36900
3	9	1116	−453,292.11841
4	305	1708	5975.73021
5	693	2484	−2724.40990
6	640	2378	6900.81223
7	690	2478	11,176.88662
8	576	2250	−10,842.84480
9	591	2280	6993.52346
10	621	2340	−23,881.16167
11	37	1172	−41,980.32481
12	582	2262	−27,614.03977
13	635	2368	12,590.34322
14	666	2430	6433.66654
15	593	2284	−21,368.79201
16	603	2304	33,900.17635
17	597	2292	24,159.88427
18	615	2328	−25,504.11354
19	649	2396	8973.45220
20	647	2392	−15,458.81667
21	11	1120	−202,417.68592
22	584	2266	20,866.93140
23	670	2438	8404.83943
24	605	2308	17,921.74157
25	627	2352	−10,725.60514
26	629	2356	22,890.11148
27	642	2382	−14,941.76960
28	585	2268	−18,343.33486
29	630	2358	−22,761.90145
30	661	2420	−9882.55575
Intercept	178.61028		

**Table 14:** Chemometric details of the variable selection/decorrelation procedure carried out by SELECT, corresponding to the optimal OLS regression model developed from column auto-scaled NIR spectra. This model is proposed to predict the TPC content of extra virgin, refined, and virgin olive oils.

Order of Selection	Predictor (Original) Index	Wavelength (nm)	Correlation Coefficient
1	210	1518	91,715.98370
2	459	2016	−19,771.41183
3	504	2106	75,503.16798
4	279	1656	−136,266.77356
5	41	1180	140,228.33992
6	494	2086	−78,991.92309
7	522	2142	−31,354.37443
8	171	1440	70,302.98905
9	178	1454	−541,936.28613
10	529	2156	34,012.60207
11	180	1458	−587,689.37666
12	546	2190	−81,746.44220
13	497	2092	−451,381.06447
14	650	2398	7408.86801
15	533	2164	172,347.35998
16	67	1232	114,433.12526
17	526	2150	−89,944.76714
18	40	1178	−420,629.27826
Intercept	739.44489		

**Table 15:** Statistical characteristics of the developed NIR and SELECT-OLS models for quantifying phenolic compounds in various olive oils with HTyr supplementation and deep frying.

Response (Phenolic Compound)	SDE	MAE	R	LOORV%	LOORSD	LOOEV%	LOOMPE
Hydroxytyrosol	19.51	13.97	0.98	5.00	21.10	95.00	15.69
Tyrosol	10.28	7.46	0.98	4.75	11.07	95.25	8.47
Caffeic acid	4.58	3.19	0.98	4.57	4.87	95.43	3.64
Oleocanthal	9.66	6.82	0.94	20.93	11.66	79.07	9.18
Oleacein	18.59	12.74	0.94	18.97	21.13	81.03	16.71
Homovanillic acid	3.89	2.67	0.96	10.26	4.36	89.74	3.26
Pinoresinol	9.66	6.51	0.91	27.98	11.05	72.02	8.57
OAOAH	14.97	10.30	0.95	17.96	17.11	82.04	13.56
LAOAH	62.26	43.37	0.94	20.07	70.07	79.93	56.36
TPC	97.81	69.87	0.96	9.86	107.56	90.14	82.38
Concentration of Response (mg/kg)	Min			Max			
Hydroxytyrosol	0			389.49			
Tyrosol	0			215.50			
Caffeic acid	0			90.99			
Oleocanthal	0			112.01			
Oleacein	0			199.34			
Homovanillic acid	0			60.21			
Pinoresinol	0			109.11			
OAOAH	0			176.65			
LAOAH	0			686.59			
TPC	3.89			1683.03			

SDE: Standard deviation of error; MAE: Mean absolute error; Multiple correlation coefficient (R); LOORV: LOO Residual variance; LOORSD: LOO Residual standard deviation; LOOEV: LOO Explained variance; LOOMPE: LOO mean prediction error. Oleocanthal: decarboxymethyl ligstroside aglycone, dialdehyde form; oleacein: decarboxymethyl oleuropein aglycone, dialdehyde form; OAOAH: oleuropein aglycone, oxidized aldehyde and hydroxylic form; LAOAH: ligstroside aglycone, oxidized aldehyde and hydroxylic form.

## Data Availability

Data will be made available upon reasonable request.
